# The Use of Infrapatellar Fat Pad-Derived Mesenchymal Stem Cells in Articular Cartilage Regeneration: A Review

**DOI:** 10.3390/ijms22179215

**Published:** 2021-08-26

**Authors:** Parviz Vahedi, Rana Moghaddamshahabi, Thomas J. Webster, Ayse Ceren Calikoglu Koyuncu, Elham Ahmadian, Wasim S. Khan, Ali Jimale Mohamed, Aziz Eftekhari

**Affiliations:** 1 Department of Anatomical Sciences, Maragheh University of Medical Sciences, Maragheh 78151-55158, Iran; pa.vahedi48@gmail.com; 2 Faculty of Pharmacy, Eastern Mediterranean University, Famagusta 99628, North Cyprus, Turkey; Moghaddamshahabi@yahoo.com; 3 Department of Chemical Engineering, Northeastern University, 360 Huntington Avenue, Boston, MA 02115, USA; websterthomas02@gmail.com; 4 Materials and Metallurgical Engineering Department, Faculty of Technology, Marmara University, Istanbul 34722, Turkey; aysecerenkoyuncu@gmail.com; 5 Center for Nanotechnology & Biomaterials Application and Research (NBUAM), Marmara University, Istanbul 34722, Turkey; 6 Kidney Research Center, Tabriz University of Medical Sciences, Tabriz 51666-15731, Iran; ahmadian.elham@yahoo.com; 7 Division of Trauma & Orthopaedic Surgery, Addenbrooke’s Hospital, University of Cambridge, Cambridge CB2 0QQ, UK; 8 Department of Pharmacology, Faculty of Medicine, Somali National University, Mogadishu 801, Somalia; gelyac2@gmail.com; 9 Department of Toxicology and Pharmacology, Maragheh University of Medical Sciences, Maragheh 78151-55158, Iran; 10 Department of Synthesis and Characterization of Polymers, Polymer Institute, Slovak Academy of Sciences (SAS), Dúbravská cesta, 9, 845 41 Bratislava, Slovakia

**Keywords:** infrapatellar fat pad, mesenchymal stem cells, adipose tissue, osteoarthritis, articular cartilage

## Abstract

Cartilage is frequently damaged with a limited capacity for repair. Current treatment strategies are insufficient as they form fibrocartilage as opposed to hyaline cartilage, and do not prevent the progression of degenerative changes. There is increasing interest in the use of autologous mesenchymal stem cells (MSC) for tissue regeneration. MSCs that are used to treat articular cartilage defects must not only present a robust cartilaginous production capacity, but they also must not cause morbidity at the harvest site. In addition, they should be easy to isolate from the tissue and expand in culture without terminal differentiation. The source of MSCs is one of the most important factors that may affect treatment. The infrapatellar fat pad (IPFP) acts as an important reservoir for MSC and is located in the anterior compartment of the knee joint in the extra-synovial area. The IPFP is a rich source of MSCs, and in this review, we discuss studies that demonstrate that these cells have shown many advantages over other tissues in terms of ease of isolation, expansion, and chondrogenic differentiation. Future studies in articular cartilage repair strategies and suitable extraction as well as cell culture methods will extend the therapeutical application of IPFP-derived MSCs into additional orthopedic fields, such as osteoarthritis. This review provides the latest research concerning the use of IPFP-derived MSCs in the treatment of articular cartilage damage, providing critical information for the field to grow.

## 1. Introduction

### 1.1. Articular Cartilage

Articular cartilage is a specialized connective tissue that lacks blood vessels, nerves, and lymphatic tissue. Consequently, cartilage tissue has limited capacity for repair and the progression of focal cartilage defects leads to more generalized degenerative changes or osteoarthritis [1]. Articular cartilage damage is a disabling disease characterized by fibrillation and subsequent destruction of the articular cartilage surface, frequently including subchondral bone damage inducing more generalized changes [2]. The adjoining synovium in articular cartilage disease includes biomarkers for significant inflammation, including Substance P, which further stimulates a local inflammatory response [3,4]. Consequently, oedema and inflammation of the infrapatellar fat pad (IPFP) and the synovial membrane cause the progression of osteoarthritis as well as articular cartilage loss that often necessitates full joint replacement. Furthermore, the synovial membrane and IPFP may interact with each other, affecting the development and progression of osteoarthritis [4,5].

### 1.2. Past and Current Articular Cartilage Treatments

Cartilage defects cause a significant disease burden and a previous study indicated that more than of 60% of knees undergoing arthroscopy have articular defects [6]. If these defects are left untreated, or managed suboptimally, they lead to the progression of more widespread degenerative changes. There are several methods for treating articular cartilage defects depending on the anatomical location, extent, shape, and depth of the cartilage defect, and age of the patient [7] (Table 1). The operative treatment techniques mentioned in Table 1 result in the formation of the less desirable fibrocartilage, or hyaline-like cartilage, as opposed to hyaline cartilage. Fibrocartilage has suboptimal biomechanical properties and does not prevent the progression of the degenerative changes of osteoarthritis [8]. Studies on cell-based techniques, including Autologous Chondrocyte Implantation (ACI) and Matrix-Assisted ACI (MACI), have highlighted the disadvantages of using fully differentiated chondrocytes, such as their difficulty in extraction, isolation, expansion, and growth in vivo after implantation. It is of the highest importance to find alternative cell sources.

### 1.3. Cell-Based Therapies

Recent advances in developing therapeutic strategies for treating articular cartilage defects [17,18] have focused on stem cell therapies and tissue regeneration to prevent the progression to osteoarthritis [19]. Stem cells have shown superiority in treating articular cartilage defects due to their ease of isolation, expansion, and culture in preliminary studies, and are a promising method for promoting articular cartilage regeneration [20]. Mesenchymal stem cells (MSCs), in contrast to autologous chondrocytes, possess a greater capacity to expand in vitro [19]. The use of MSCs does not raise ethical concerns, as is the case with embryonic stem cells. Many questions need to be answered before stem cells are routinely used for cartilage defects, including the optimal stem cell source, an optimal extraction, isolation, and expansion protocol, the need for scaffolds, and cost. Although adipose tissue-derived MSCs have a lower chondrogenic capacity in comparison with bone marrow mesenchymal stem cells [21], they can be harvested in a less invasive and cost-effective manner with liposuction. Due to this easier access, the therapeutic application of adipose tissue-derived MSCs is increasing [22]. For all of the above reasons, the IPFP has become an area of high interest in regenerative medicine since it stores MSC.

Despite the promise of the IPFP as a source of MSC for regenerating articular cartilage, very few reviews exist on this subject. In this review, for the first time, the authors detail several IPFP cell-based therapies, their current progress towards healing articular cartilage damage, and the most recent advances in the use of IPFP-derived MSCs for cartilage repair.

## 2. Advantages of Adipose Tissue-Derived MSCs for Articular Cartilage Repair

The presence of MSCs in adipose tissue from liposuction was first reported in 2001 by Zuck et al. [23]. Adipose tissue-derived MSCs have also demonstrated a greater ability to differentiate into other lineages in pre-clinical studies compared to umbilical cord stem cells [2,24]. Adipose tissue is one of the most easily accessible tissues for the extraction of MSCs and is often discarded after liposuction. Since increased BMI and adipose tissue content are related to articular cartilage damage, removal of adipose tissue through liposuction and subsequent isolation of adipose tissue-derived MSCs can be well suited for treating articular cartilage damage [25,26].

Due to the potentially wide applications of MSCs in regenerative medicine, it is essential to have access to a reliable and reproducible MSC source. Adipose tissue-derived MSCs are feasible and promising candidates for cell-based therapies [27], and they can be differentiated into adipose tissue, bone, cartilage, and muscle [28,29]. Studies have confirmed chondrogenic differentiation of human adipose tissue-derived MSC pellet cultures by the expression of target tissue markers [30]. After harvesting of adipose tissue using liposuction aspiration or needle biopsy, adipose tissue-derived MSCs were isolated from adipose tissue. The tissue was washed with phosphate-buffered saline and a penicillin/streptomycin solution before being minced. To further dissolve any adipose tissue clumps or aggregates, the adipose tissue was pipetted up and down numerous times to facilitate mechanical disruption of the extracellular matrix. The tissue was then placed in a plate of sterile tissue culture dishes and 0.05% of a collagenase digestion buffer for tissue digestion after the debris was removed. The supernatant was aspirated after collagenase inactivation with K-NAC medium supplemented and 10% fetal calf serum (FCS), and the cell pellet was resuspended in a K-NAC medium supplemented with 10% FCS. Following centrifugation, the cell suspension was blended and filtered using a 100-μm cell strainer. Lastly, cell pellets were plated onto a tissue culture plate and cultured in an incubator at 37 °C with 5% CO_2_ [22]. Such processes are now commonly used for ADSC isolation and can be used for articular cartilage repair.

Over the last two decades, stem cell-based therapies using adipose tissue-derived MSCs have been expanding, as supported by their strong therapeutic potential. Studies have reported that the effect of stem cells derived from different tissues differ according to the site of extraction [31]. Adipose tissue is easier to access than other tissues and obtaining MSCs from this tissue is less invasive [32]. Compared to bone marrow, the process of harvesting tissue from adipose tissue is less invasive, and studies suggest a greater cell yield per unit of tissue as well [33]. Studies suggest that adipose tissue-derived MSCs have a smaller cell body than bone marrow-derived MSCs and have different gene expression and cell surface receptors. Commonly used markers include CD90, CD44, CD29, CD105, CD13, CD34, CD73, CD166, CD10, CD49e, and CD59, which are all positive, while CD31, CD45, CD14, CD11b, CD19, CD56, and CD146 are all negative in adipose tissue-derived MSCs. In addition, the positive expression of HLA-ABC and STRO-1 as well as the negative expression of HLA-DR are also features of adipose tissue-derived MSCs [34]. ADSCs can all be passaged in vitro up to passage 10 with no karyotype abnormalities detected [35]. Unlike bone marrow-derived MSCs, the number, viability, and proliferation capacity of ADSCs do not appear to be related to patient age.

Despite the important advantage of adipose tissue-derived MSCs in that they are easier to harvest and isolate, and their increased ability to proliferate and differentiate into chondrocytes, an incomplete understanding of the processes and mechanisms of their differentiation have limited their clinical applications [24]. Studies have reported important differences between various MSC sources, and this has implications on the choice of cells for articular cartilage regeneration [36,37]. MSCs from various tissues vary in their proliferation and differentiation properties (Table 2). An important challenge with adipose tissue-derived MSCs is the creation of fibrous and hypertrophic cartilage instead of articular hyaline cartilage [36].

## 3. IPFP-Derived MSCs Used in Cartilage Repair 

IPFP-derived MSCs are a subset of MSCs and may have higher cartilage regeneration potential than other MSCs due to their proximity to the knee joint and similarity to subcutaneous adipose tissue cells; they are also more easily accessible than other MSC tissues [39]. The IPFP is intracapsular and extra-synovial, and is located between the patellar tendon, the femoral condyle, and the tibial plateau [40]. The IPFP is a less invasive source of MSCs that can be easily accessed with less morbidity arthroscopically [40]. Obtaining subcutaneous fat by liposuction can obtain a large amount of adipose tissue, however, this can be associated with complications such as skin necrosis, scarring, hematoma, allergic reactions to drugs, temporary bruising, numbness, and nerve injury [41,42].

The cellular environment and adjacent tissues alter cell gene expression [43]. The IPFP is adjacent to the synovial membrane and the synovial fluid as well as articular cartilage, and is potentially influenced by this anatomical location in terms of stem cell differentiation [44]. Additionally, the IPFP cells are likely to be positively affected by biomolecules in the synovial membrane and synovial fluid including TGF-β1, vitamin C, and FGF [43]. Studies show that cartilage-derived morphogenic protein-1 (CDMP-1) and osteogenic protein-1 (OP-1) are more highly expressed in the synovial tissue than in articular cartilage tissue [33], and this can certainly affect IPFP-derived MSC numbers, differentiation, and function [45,46].

The general characteristics of IPFP and adipose tissue-derived MSCs are similar, but the differentiation capacity of IPFP-derived MSCs compared to a chondrogenic and osteogenic lineage is higher than that of adipose tissue-derived cells. SOX-9, collagen type II, and aggrecan gene expression are higher in IPFP-derived MSCs than adipose tissue-derived MSCs, which may explain their more optimal use for articular cartilage regeneration applications [47].

IPFP, and specifically the macrophages from the tissue, can be candidates for the future treatment of cartilage defects and chondrogenesis [42]. Importantly, polarization of macrophages to pro-inflammatory (M1) or anti-inflammatory (M2) phenotypes led to an upsurge in TGF-β (a pro-chondrogenic and anti-inflammatory cytokine) that may play a key role in the differentiation of MSCs and subsequent cartilage repair.

In summary, the use of IPFP-derived MSCs to regenerate articular cartilage has led to encouraging results. The collaboration of researchers from many disciplines and different fields will help address current challenges concerning the therapeutic role of IPFP-derived MSCs, including the best isolation process, and should pave the way for the improved treatment of articular cartilage. Future detailed studies regarding articular cartilage repair strategies focusing on suitable material and methods may eventually extend the application of IPFP-derived MSCs to a future therapeutic role in osteoarthritis.

Many MSCs when injected into the body migrate away from damaged tissue to healthy tissue and a suitable carrier may be needed to “anchor” the MSCs in place [48]. The identification of the optimal cell source as well as the optimal carrier material for these cells plays an important role in the success of stem cell-based regenerative strategies (Table 3).

The studies mentioned above and other important studies in the literature that used IPFP-derived MSCs for regenerative applications in chondrogenesis are listed in Table 4.

## 4. Clinical Applications of IPFP-Derived MSCs for Articular Cartilage Repair

From a therapeutic perspective, clinical studies have already been conducted using adipose tissue and IPFP-derived MSCs (Table 5) [66]. In a clinical study, an average of 1.89 × 10^6^ MSCs isolated from osteoarthritic knees were combined with 3 mL of platelet-rich plasma and placed in a novel biological scaffold. Twenty five patients (eight men and seventeen women) with knee osteoarthritis received these percutaneously administered injections combined with arthroscopic debridement [45,66]. The constructs, along with platelet-rich plasma, were transplanted into articular cartilage defects to facilitate articular cartilage repair. This study showed that at a one-year follow-up, patient knee pain grading scores, including the Tegner activity scale, the mean Lysholm, and visual analog scale (VAS), all significantly improved, with 92% of 25 patients showing an improvement in these scores. 

The use of IPFP-derived MSCs in such clinical studies led our group to use adipose-derived MSCs with polycaprolactone to form a 3D “cartilage-like” chondrogenic structure which was then transplanted into small cartilage defects in the knees of sheep. Tissue was isolated from the IPFP of five male sheep and after 6 months, amorphous proliferative tissue was regenerated in the defect area. Real time RT-PCR analysis proved collagen protein expression as well as greater cartilage regeneration compared to controls [69,70]. In this study, the ideal size of a cartilage defect was designated as 1 mm in depth and 4 mm in diameter. A 3D IPFP-derived MSC/polycaprolactone structure was directly transplanted into the defects. Within 6 months, the 3D graft improved cartilage formation and regenerated the cartilage without any inflammatory reaction and in the newly formed cartilage tissue, polygonal chondrocyte clusters were shown. As a result, histological analysis, including staining of safranin O, morphological assays, and the expression of type I and type II collagen, confirmed hyaline-like cartilage formation but without integration into the surrounding cartilage [68].

One of the most prominent clinical studies to date [68] described the safety and effects of an intra-articular injection of adipose tissue-derived MSCs prepared from abdominal subcutaneous fat obtained by liposuction [71] on 18 patients with knee osteoarthritis. At the final six-month follow-up, results demonstrated the formation of new cartilage in the knee joint at the medial femoral and tibial condyles, and a decrease in cartilage defect size using Magnetic Resonance Imaging (MRI).

Dufrane et al. used human adipose tissue-derived MSCs with a demineralized bone matrix to generate a 3D, bone-like construction and transplanted them into large bone defects in six patients. After three months, bone growth was observed and the anatomy and function of the defect was restored without any adverse effects [72]. In another study, human adipose tissue-derived MSCs in combination with platelet-rich plasma, hyaluronic acid, and CaCl_2_ were injected into the knees of osteoarthritis patients. Bone and cartilage regeneration were observed over three months and showed increased cartilage volume and bone regeneration [67]. Histological assessment confirmed the formation of hyaline-like cartilage integrated into subchondral bone. Poor integration of cartilage into bone is one of the most common problems in articular cartilage repair [67,73]. Furthermore, the efficiency and safety of an intra-articular injection of adipose tissue-derived MSCs in osteoarthritis patients was also investigated [67].

In summary, these studies in the literature not only support the use of IPFP-derived MSC for osteoarthritis therapy and articular cartilage regeneration, but they also provide encouraging clinical results. There are, however, some limitations to the use of IPFP-derived MSC in clinical applications; in human models, researchers typically obtain IPFP tissues from patients that have been diagnosed with osteoarthritis, which affects the function and differentiation activity of IPFP-derived MSC [63,64].

MSCs have shown promise in regenerative medicine and tissue engineering. In the field of regenerative medicine, cell therapy methods involving direct injection, cell seeding on scaffolds, and transplantation of stem cells to the site of the defect have been applied [67]. Much of the evidence in this review supports the use of MSCs together with scaffolds comprising a proper biomimetic structure [74].

Before using MSCs, it is necessary to choose the specific tissue and MSCs for more efficient cartilage regeneration [75]. Identifying the anatomical location of the tissue to harvest MSCs from and the characterization methods to use for the MSCs are promising future directions for this field [76]. Adipose-derived MSCs that are used in cell therapy are obtained mostly from subcutaneous adipose tissue by liposuction. Previous studies have demonstrated that the mechanical disruption associated with liposuction may adversely affect MSCs and may reduce their proliferative capacity [51,77,78]. The IPFP is a rich source of MSCs for articular cartilage regeneration and contains a high number of chondroprogenitor cells [45,72]. Evidence suggests that the IPFP location [79] adjacent to the synovial membrane and fluid potentiates its use for articular cartilage regeneration [80,81,82]. The literature suggests that the proliferation and differentiation potential of IPFP-derived MSCs is independent of age, while MSCs from other sources generally undergo an age-related decline in potential [66]. Recently, IPFP-derived MSCs have been used in cell-based therapies for healing cartilage defects, as reported by Ashton et al. [83].

**Figure 1:**
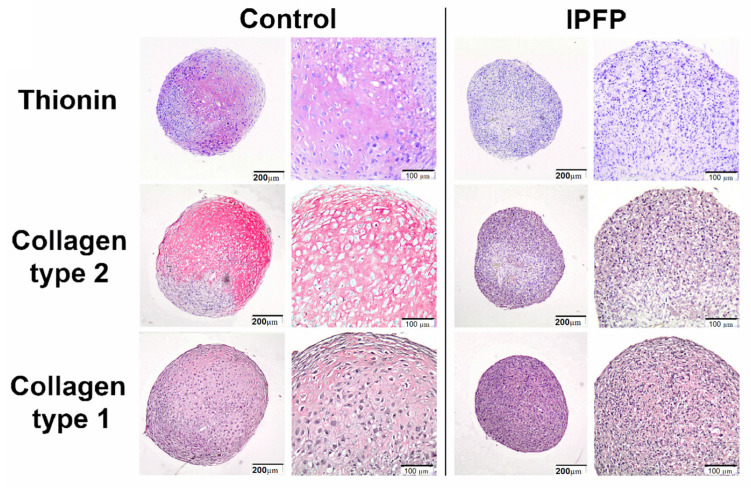
IPFP inhibits chondrogenic differentiation of MSCs. Immunohistochemically stained images of pellets from MSC donors for glycosaminoglycans with thionin or collagen type II and collagen type I showing IPFP decreased thionin and collagen type II, whereas collagen type I staining was not affected. Reproduced with permission [65]. Copyright 2015, Elsevier.

The self-repair capacity of hyaline cartilage is very limited. Although small subchondral defects may spontaneously repair with the production of hyaline cartilage, larger chondral defects generally heal with fibrocartilage [39]. Fibrocartilage is histochemically and biomechanically inferior to normal hyaline cartilage. Cartilage defects frequently progress to more generalized osteoarthritic changes [84]. Interestingly, a study has shown that hyaline cartilage may not be the definitive repair tissue in the healing of articular cartilage defects [85], and it may be produced at an intermediary stage; in the process of ossification, hyaline cartilage is formed first. So, detailed studies are necessary for articular cartilage repair. In addition, the use of suitable harvesting methods would improve the efficacy of IPFP-derived MSCs in their future therapeutic role in regenerative medicine [86]. Wei et al. reported that the immunohistochemical analysis of IPFP and adipose tissue indicated that IPFP contains more macrophages [65] (Figure 1). Previous studies have shown that pro-inflammatory macrophages prevent chondrogenesis and induce MSCs to produce a fibrocartilage matrix [87,88]. Therefore, due to the various challenges and opinions regarding the therapeutic role of IPFP-derived MSCs, further studies are needed [21].

## 5. Conclusions

In summary, the use of IPFP-derived MSCs to regenerate articular cartilage has achieved better results than MSCs from other sources. The collaboration of researchers from many disciplines and different fields will help solve current challenges facing the therapeutic applications of IPFP-derived MSCs and may create new therapies for the treatment of articular cartilage defects.

## Figures and Tables

**Table 1:** Non-operative and operative treatment techniques for articular cartilage defects.

Technique	Effect	Reference
Non-operative Methods	Pharmacotherapy drugs (steroidal and non-steroid anti-inflammatory drugs, glucosamine, chondroitin sulphates, etc.) to control symptoms	[9]
Abrasion Arthroplasty	Creation of a rough surface in the damaged area in order to form fibrocartilage (subchondral bone is not directly accessed)	[10]
Arthroscopic Debridement	Removal of the damaged part of the tissue, allowing the subchondral bone to initiate the healing process	[11]
Autologous Chondrocyte Implantation (ACI)	Transplantation of isolated and expanded chondrocytes from healthy cartilage to the defected area	[12]
Chondroplasty	Utilizes laser or radiofrequency-based probes to smooth the damaged edges of cartilage	[13]
Matrix-assisted ACI (MACI)	Isolated and expanded autologous chondrocytes are combined with a scaffold which is implanted into the defect site	[14]
Microfracture (subchondral drilling)	Subchondral bone is stimulated by drilling, allowing the bone marrow MSCs to migrate to the damaged area and form fibrocartilage	[15]
Mosaicplasty	Osteochondral autografts or allografts are transferred from a donor site to the defect site	[16]

**Table 2:** A summary of MSC properties with respect to their isolation, proliferation, and differentiation [37,38].

	Adipose Tissue-Derived MSCs	Bone Marrow-Derived MSCs	Umbilical Cord-Derived MSCs
Ease of Harvest and Isolation	+++	+	++
Amounts of Tissue Obtained	+++	++	+
Capacity for Proliferation and Colony Formation	++	+	+++
Maintenance of Function Irrespective of Donor Age	++	+	+++
Suitability for Soft Tissue Regeneration (e.g., skin, cartilage, etc.)	+++	++	+
Suitability for Hard Tissue Re-generation (e.g., bone, tooth, etc.)	+	++	+++

+ weak, ++ moderate, +++ strong.

**Table 3:** Some important studies on IPFP-derived MSCs for articular cartilage repair that use a carrier.

Cell Source	Carrier	Outcome	Reference
Porcine IPFP-derived MSCs	Agarose hydrogels	Chondrogenic differentiation of cells in vitro were shown by histochemistry and biochemical analyses of glycosaminoglycans, as well as mechanical tests within 6 weeks	[49]
Porcine IPFP-derived MSCs	Fibrin hydrogels incorporated with TGF-β1-loaded gelatin microspheres	In vitro chondrogenesis of cells promoted for 21 days, as demonstrated by histochemistry and biochemical analyses of glycosaminoglycans	[50]
Human IPFP-derived MSCs	3D-printed chitosan scaffolds	Cartilage-like tissue formed on the constructs after 4 weeks of culture, as demonstrated by immunohistochemistry analyses and collagen type II, aggrecan, and SOX9 gene expression	[51]
Human IPFP-derived MSCs	Acellular dermal matrix (ADM) from rat dermis	Differentiation towards a hyaline-like cartilage phenotype on the constructs as proven by the expression of collagen type II, aggrecan, and SOX9 over 4 weeks in vitro	[52]
Sheep IPFP-derived MSCs	Nanofibrous polycaprolactone (PCL) scaffolds	Constructs promoted hyaline-like cartilage formation in vivo, evaluated by SOX9 and aggrecan expression for over 6 months	[53]
IPFP-derived MSCs	Scaffold-free 3D pellet culture	Constructs provided osteochondral regeneration in a rat osteochondral defect model within 12 weeks	[54]
Human IPFP-derived MSCs	Scaffold-free 3D pellet culture	Constructs integrated well into the femoral condyle, presenting hyaline cartilage features as indicated by SOX9 and COL2A1 gene expression in vitro and in vivo	[55]
Rabbit IPFP-derived MSCs	3D gelatin-based biomimetic scaffold	Chondrogenic tissue formation as evidenced by collagen type II, aggrecan, and SOX9 expression in vitro for 3 weeks	[56]

**Table 4:** Studies on IPFP-derived MSC proliferation and chondrogenic differentiation.

Tissue Donor	Assay	Results	References
Articular Cartilage in the Knee in a Porcine Model (*n* = 3) (4 months old)	Cells harvested and population doubling time	FGF-2 increased IPFP-derived MSC proliferation	[57]
Pigsty (*n* = 2) (5 months old)	Cell calculation	ECM development promoted IPFP-derived MSC proliferation and maintained stem cell morphology	[58]
Patients for Sectional Surgical Removal of a Meniscus (*n* = 15) (26–68 years old)	Cell production and population doubling time (PDT)	Transforming growth factor beta (TFP) completion considerably promoted the proliferation rate of the meniscus of joint cells, IPFP-derived MSC, and SDSCs, but less than that of adipose tissue-derived MSCs	[59]
Adolescent Patients with Anterior Cruciate Ligament (ACL) Trauma for Regeneration (*n* = 4) (17.2 (SD 0.7) years old)	Cell calculation	IPFP-derived MSC has less proliferative capacity than SDSCs but more than ScASCs	[60]
Osteoarthritis in Old Patients for Whole Knee Arthroplasty (*n* = 4) (70.5 (SD 9.2) years old)	Cell counting	IPFP-derived MSC have more proliferative capacity compared with SDSCs but more than other cases	[61]
IPFP-derived MSCs from Sheep Knees (*n* = 5) (10,12,14,16,18 months old)	Transplantation of IPFP-ASCs	Hyaline cartilage-like formed in some cartilage defects	[62]
IPFP-derived MSCs from Sheep Knees (*n* = 5) (12 months old)	Assay of doubling time ASC	Maintain proliferation potential and fibroblastic-like morphology of IPFP-derived MSCs during different passages	[53,62]
IPFP-derived MSCs Patients (*n* = 7) (74 years old)	Isolation of IPFP-stem cells and cell calculation	The extracellular matrix fragments in suspension prevented the calculation of cells	[63]
IPFP, Patients (*n* = 5) (50 years old)	Yielding of IPFP by liposuction and isolation of stem cells	IPFP-derived MSCs are an important alternative source for adipose-derived cells	[64]
IPFP-derived MSCs, Patients (*n*= 8 Men and 17 Women) (30 years old)	Assaying the stem cell injection	IPFP-derived MSCs treatment with intra-articular injections is effective and reduces pain	[45]
Macrophages from Adipose tissue, Patients with Osteoarthritis (60 years old)	Harvesting of macrophages from IPFP	The macrophages from IPFP can change articular cartilage-based stem cell therapy	[65]

**Table 5:** Clinical studies on the use of MSCs for articular cartilage regeneration.

Study	Cases	Outcome	Prospects	Reference
Autologous adipose tissue-derived MSCs injected into the hips of patients, in combination with hyaluronic acid (HA), platelet rich plasma (PRP), and CaCl_2_	Osteonecrosis of the hip femoral head: 1 female (age 29) and 1 male (age 47) Osteoarthritis: 2 females (ages 70 and 79)	Bone formation in osteonecrosis patients and cartilage formation in osteoarthritis patients within 1–3 months, as evidenced by MRI	ACS + HA + PRP + CaCl_2_ presents a slightly invasive therapy for osteonecrosis and osteoarthritis	[67]
Percutaneous injection of IPFP-derived MSCs into arthritic knees	Knee osteoarthritis: 8 male and 17 female patients (age range, 34–69)	Pain reduction and improvement in function in patients with knee osteoarthritis within 3–18 months	For knee osteoarthritis, this could be an effective treatment after optimization of some parameters such as cell and injection numbers	[45]
Intra-articular injection of autologous adipose tissue-derived MSCs into arthritic knees	Knee osteoarthritis: 15 female and 3 male patients (age range, 18–75)	Radiological, arthroscopic, and histological analyses demonstrated hyaline-like cartilage formation, resulting in pain reduction and improvement in function 6 months after the injection into osteoarthritic knees	Large randomized clinical trials for the treatment of osteoarthritis	[68]
